# Indents

**Published:** 1901-05-15

**Authors:** 


### INDENTS.

**☞ Brief Articles of Technical or Scientific Value will receive notice and comment. Contributions invited.**

VARNISH FOR CEMENT FILLINGS.—Dr. Daboll recommends a chloroform solution of Canada Balsam to protect cement fillings for a few hours while hardening. He also uses it to protect sensitive cavities from irritating filling material.—*Cosmos.*

A COUNTER-IRRITANT.—Mix together ginger, red pepper and mustard, and sprinkle it on a split raisin and roast it, and then apply to gum over affected tooth. It is more effective than the roasted raisin alone.—Dr. Roberts, in *International Journal.*

TRANSFORMING LOGAN CROWNS.—Dr. A. P. Burkhart, of Buffalo, suggests that more artistic results will be obtained when setting Logan crowns if the crowns are ground to match adjacent teeth. The tooth can be polished with sandpaper and cuttle-fish paper disks after grinding so that its lustre may be maintained.—*Cosmos.*

TO DISINFECT A PUTRID ROOT CANAL.—Dr. H. Prinz suggests that sodium per oxid be substituted for sodium bicarbonate to counteract the sulphuric acid used. The chemical reaction produces free hydrogen per oxid and sodium sulphate. The per oxid of hydrogen bleaches, and by its evenness mechanically assists in removing debris from the canal, while the sodium sulphate saponifies and sterilizes the root canal, without danger of peridental irritation.

HIGH-PRICED INSTRUMENTS.—I never buy a really good instrument, which will help me in my work, that I do not feel that it would be cheap at any price. Dental supply houses are as necessary to us as the army commissary is to the army. We should have sympathetic relations with those who endeavor to supply our needs; the advantages will surely be mutually helpful. Sometimes the rain falls so steadily and gently about us that we fail to appreciate its blessings.—S. G. Perry, April *Items of Interest*.

PULP ANODYNE.—Dr. Wessels, in the April *Dental Brief*, gives the following anodyne formula: Take of camphor, thirty grains; oil of cloves, one-half dram; tincture of opium, eight drops; chloroform, two ounces; ether, six drams; alcohol, one ounce, and apply on cotton and cover with Sandaraced cotton or temporary gutta-percha. Also the following for a dressing in pulpless teeth in the intervals of treatment: Take carbolic acid, four drams; thymol, two drams, and one dram each of oil of cassia, oil of cloves and glycerine. Seal into cavity with gutta-percha.

PROPER SPHERE OF THE ALL-GOLD CROWN.—Dr. T. A. Black, in the *Pacific Dental Gazette*, says: The all-gold crown is one of the grandest inventions of modern dentistry, but its legitimate place is not in conspicuous position in the mouth, except where it is necessary to make useful, roots which otherwise must be sacrificed. To have the continuity of a handsome set of teeth broken by the insertion of a gold crown anywhere between the second bicuspids, especially of the upper jaw, is unjustifiable, and offensive to good taste. A conspicuously crowned tooth is not only offensive in itself, but it discredits other contiguous teeth.

Making Inlay Matrix.—Dr. Broomel, in April *Cosmos*, suggests the use of a small rubber water bag for swaging platinum into a plaster matrix which is confined in one end of metallic cylinder, to which is fitted a metal plunger. The apparatus is similar to the Parker shot swaging apparatus. The advantages claimed are that an accurate impression may be made in plaster of Paris, into which the platinum may be driven by the yielding water bag under pressure from force applied to the plunger by mallet blows or continuous pressure, thus securing accurate adaptation. The work can be done without the presence of the patient.

Ethics.—Dr. S. G. Perry says, one can have more knowledge than another, but one can not have more honesty than another. Knowledge is power, and a learned profession may have more power than an unlearned one, but a dishonest use of knowledge is fatal. A business honestly conducted can not be deprived of men's respect. The professional man really sells his services to the highest bidder, and justifies his fees on the ground that he is entitled to them or he would not get them. He assumes that a discriminating public knows what it wants. Learning and labor are brothers. Learning may be first, but there is nothing degrading in honest labor. And honest business methods are essential in the full utilization of both.—April *Items of Interest*.

Polishing Aluminum Plates.—It is a very difficult matter to obtain a satisfactory finish to an aluminum plate. No matter what means are used in polishing, there is always a dull lead color, so unlike the bright appearance of this useful metal in its pure state. This can be overcome, after the final polish has been given with brush wheels, etc., by coating the plate with a strong solution of caustic soda. Use a pledget of cotton on pliers dipped in the solution, coating the metal freely on both sides, allowing it to remain two or three minutes, then wash thoroughly with soap and water. If there still be dark spots, apply again to those places until they disappear. The solution will not affect the rubber attachments and will enhance the appearance of the finished plate 50 percent.—H. F. Naumann, *Items of Interest*.

Trade Journals.—Trade journals, because of their wide circulation, made possible by trade interests, have carried the best thoughts of our leading men to the uttermost parts of the earth, changing us from the formal, bewigged and unapproachable wiseacres of the beginning of the nineteenth century to the vital, adaptive professional man of the opening of the twentieth century. As the profession has improved, so has the trade journal. The production of a really live and good professional journal is a business like any other calling, and requires business management and supervision, to say nothing of financial support; it will not run itself, no matter how professional or independent it may be. This will at least suggest the inconsistency of sneering at trade conditions. After all, trade influences and interest sink into insignificance when a journal is conducted by a man of knowledge, sound in practice, and whose devotion to the best interests of his profession is tireless in season and out. Our professional dignity and literature will not suffer in such hands.—S. G. Perry.

Emergency Crowns.—Dr. L. C. Jones describes in the April *Cosmos* a crown which can be expeditiously made where opportunity may not allow a more substantial substitute. Select a proper tooth, such as is used in vulcanite work. Flatten the end of a piece of round German silver wire, and notche the edges of the flattened end so that it will fit in between the tooth pins, which are then drawn together so as to hold the German silver post tightly to the porcelain tooth. It is then fitted to the root, previously prepared, and held in position while some warm wax is crowded in behind it so as to get an impression of the root end and also form a contour for the crown. It is then removed from the tooth and invested in plaster. When the plaster has set melt out the wax with boiling water, and dry over a moderate flame. When dry moisten the metal parts with muriate of zinc flux, and place a few pieces of fusible metal on the crown, melt the alloy with a blast from the blowpipe, and just as it begins to harden moisten the finger and press the allow in every part of the matrix. Remove from the investment, trim to shape, polish and set as usual. To keep the end of the root dry and free from the gum, pack it with Gilbert's temporary stopping.

